# The Landauer Principle: Re-Formulation of the Second Thermodynamics Law or a Step to Great Unification?

**DOI:** 10.3390/e21100918

**Published:** 2019-09-20

**Authors:** Edward Bormashenko

**Affiliations:** Department of Chemical Engineering, Biotechnology and Materials, Engineering Sciences Faculty, Ariel University, Ariel 407000, Israel; edward@ariel.ac.il; Tel.: +972-074-729-68-63

**Keywords:** Landauer principle, entropy, information, relativity, particle, field

## Abstract

The Landauer principle quantifies the thermodynamic cost of the recording/erasure of one bit of information, as it was stated by its author: “information is physical” and it has an energy equivalent. In its narrow sense, the Landauer principle states that the erasure of one bit of information requires a minimum energy cost equal to *k_B_T* ln2, where *T* is the temperature of a thermal reservoir used in the process and $k_{B}$ is Boltzmann’s constant. The Landauer principle remains highly debatable. It has been argued that, since it is not independent of the second law of thermodynamics, it is either unnecessary or insufficient as an exorcism of Maxwell’s demon. On the other hand, the Landauer principle enables the “informational” reformulation of thermodynamic laws. Thus, the Landauer principle touches the deepest physical roots of thermodynamics. Authors are invited to contribute papers devoted to the meaning, interpretation, physical roots, experimental verification and applications of the Landauer principle. Papers devoted to the quantum and relativity aspects of the Landauer principle are encouraged.

## 1. Introduction

Informational theory is usually supplied in a form that is independent of any physical embodiment. In contrast, Rolf Landauer in his papers argued that information is physical and it has an energy equivalent [1,2,3]. It may be stored in physical systems such as books and memory chips and it is transmitted by physical devices exploiting electrical or optical signals [1,2,3]. Therefore, he concluded, it must obey the laws of physics and, first and foremost, the laws of thermodynamics. The Landauer principle [1,2,3], establishing the energy equivalent of information, has remained the focus of investigations in the last decade [4,5,6,7,8,9,10]. In spite of the fact that non-equilibrium and quantum extensions of the Landauer principle were reported, the exact meaning and formulation of the principle remain obscure and both of them have been exerted in the stormy discussion [5,6,7,11,12]. The Landauer principle in its general meaning established the relation between thermodynamic and logical reversibility, and consists of two statements [7,11,12]: (1) any logically irreversible process must result in an entropy increase in the non-information-bearing degrees of freedom of the information-processing system or its environment; (2) any logically reversible process can be implemented thermodynamically reversibly. In its strict, tight and simplest meaning, the Landauer principle states that the erasure of one bit of information requires a minimum energy cost equal to *k_B_T* ln2, where *T* is the temperature of a thermal reservoir used in the process and *k* is Boltzmann’s constant [1,2,3,4,8,13]. Later, Landauer applied the principle to the *transmission* of information and re-shaped it as follows: an amount of energy equal to *k_B_T* ln2 (where *k_B_T* is the thermal noise per unit bandwidth) is needed to transmit a bit of information, and more if quantized channels are used with photon energies *hν* > *kT* [3]. The extension of the Landauer principle for the situation when multiple physical quantities (and not only energy) are conserved has been reported [13,14]. An angular momentum (spin) cost necessary for the recording/erasure of information was reported [13,14]. The generalization of the Landauer principle for indeterministic operations was addressed [15]. 

On the other hand, the Landauer principle was intensively criticized by J. D. Norton, who argued that, since it is not independent of the second law of thermodynamics, it is either unnecessary or insufficient as an exorcism of Maxwell’s demon [5,6,16,17,18]. J. Bub, in turn, suggested the asymmetry of the recording and erasure of information and stated that there is in principle no entropy cost to the acquisition of information, but the destruction of information does involve an irreducible entropy cost [19]. The Landauer principle was defended in a series of recent papers [11,12], however the discussion is far from being exhausted. In our present paper, we demonstrate the informational re-formulation of thermodynamics [20,21,22,23,24,25], which may be useful as a step for the “great unification” of physics, enabling a new glance on the fundamental physical problems. 

## 2. The Fundamental Aspects of the Landauer Principle 

### 2.1. The Landauer Principle and Relativity

The application of the Landauer principle to the relativity problems was discussed in a few of the papers [26,27]. The interrelation between the Landauer principle and the relativistic transformation of temperature was discussed in [27]. Consider a particle with energy *E* in contact (not necessarily in thermal equilibrium) with the thermal bath *T*. The energy of the particle may be transferred into the work, used for recording/erasing information within the thermal bath. According to the Landauer principle and relativity, the maximal information $I_{max},$ which may be recorded by the particle with the bath, equals:

(1) $$I_{max}=\frac{E}{k_{B}Tln2}=\frac{mc^{2}}{k_{B}Tln2}$$

where *k_B_* is the Boltzmann constant and *m* is the relativistic mass of the particle. Equation (1) appears in [28], in which the mass of one bit of information $m_{bit}=\frac{k_{B}Tln2}{c^{2}}$ is introduced. The value $I_{max}$ in turn may be seen as the maximal informational content of a relativistic particle. If the potential energy of the particle is negligible, and $\frac{v}{c}\ll c$ takes place, where *v* is the velocity of the particle, Equation (1) is re-shaped as follows:

(2) $$I_{max}=\frac{m_{0}c^{2}}{k_{B}Tln2}$$

The value $I_{max}$ supplied by Equation (2) may be interpreted as the maximal informational content of a particle *at rest*. Equation (2) is highly disputable due to the possibilities of the violation of the Landauer principle discussed in [29]. Authors are encouraged to submit papers devoted to the Landauer principle, seen in a view of relativity. 

The particle may exchange information with the medium, if *at least one bit* of information is transferred from the particle to the medium (thermal bath), thus inequality $I_{max}\geq 1$ should hold. This inequality yields:

(3) $$m_{0}\geq \frac{k_{B}Tln2}{c^{2}}$$

The particle with a rest mass smaller than ${\tilde{m}}_{0}=\frac{k_{B}Tln2}{c^{2}}$ does not transform information to the medium at the temperature of *T*. Assuming T=2.73 K (which is the temperature of the cosmic microwave background [30]), we obtain the estimation ${\tilde{m}}_{0}≅\frac{1.6\times 10^{-4}eV}{c^{2}}≅2.0\times 10^{-40}\;\mathrm{kg}$. It should be emphasized that all currently known elementary particles (including neutrino $m_{neutrino}<0.120\frac{eV}{c^{2}}$) are heavier than ${\tilde{m}}_{0}=2.0\times 10^{-40}\;\mathrm{kg}$. Particles lighter than ${\tilde{m}}_{0}=2.0\times 10^{-40}\;\mathrm{kg}$ do not transform the information to the Universe and are well expected to be undetectable. Again, possible violations of the Landauer principle should be considered [29].

### 2.2. The Landauer Principle and the Great Unification of Physics 

The Landauer principle supplies a new glance on the problem of the great unification of physics. Indeed, Equation (1) may be easily extended to fields. Consider a field (for example, an electromagnetic field) in a thermal contact (not necessarily in thermal equilibrium, as it takes place in a black body radiation problem) with surrounding *T*. The energy of the field may be used for recording/erasing information in the surrounding bodies. The maximal information to be recorded (the informational content of the field) according to the Landauer principle is given by:

(4) $$I_{max}=\frac{E_{f}}{k_{B}Tln2}$$

where *E_f_* is the energy of the field. Consider, that the physical nature of the field does not matter. If the information and the temperature are taken as basic physical quantities, Equation (4) is universal for all kinds of physical fields. The field is capable of recording/erasing the information if inequality $E_{f}>k_{B}Tln2$ takes place. The Landauer principle changes the status of temperature, usually seen as the derivative of basic physical quantities, such as energy and entropy. Contrastingly, the Landauer principle tells us that it is just the temperature that determines the possibility of recording/erasing the information, seen as a basic physical value. 

### 2.3. The Landauer Principle and the Relativistic Transformation of Temperatures

The relativistic transformation of temperatures remains a subtle and open theme, in which different expressions for this transformation were suggested [27,31,32,33,34,35]. Planck and Einstein suggested that the transformation of temperatures is governed by: $T=T_{0}\sqrt{1-\frac{u^{2}}{c^{2}}}$ [31]. In contrast, Ott suggested for the same transformation $T=\frac{T_{0}}{\sqrt{1-\frac{u^{2}}{c^{2}}}}$ [32]. It was mentioned that the relativistic transformation of temperatures works when energy is transformed under constant momentum, whereas the transformation suggested by Ott is valid when the velocity of a particle is constant [31,32]. Consider that in relativity the momentum and velocity of a particle are not proportional to one another. Thus, it was suggested that an unambiguous relativistic transformation of temperatures is impossible [33,34,35]. It was also suggested that temperature is the relativistic invariant [34].

It is reasonable to suggest that the maximal number of bits that may be recorded by a particle in the thermal bath is a relativistic invariant (as well as that the entropy is the relativistic invariant [31]). Thus, the Landauer principle and Equation (1) support the idea that temperature is transformed according to the transformation suggested by Ott, namely $=\frac{T_{0}}{\sqrt{1-\frac{u^{2}}{c^{2}}}}$.

### 2.4. The Landauer Principle and the Dark Matter Problem

The Landauer principle enables a fresh glance on the “dark matter” problem [36]. We still do not know how to explain how stars orbit in galaxies and how galaxies orbit in clusters. A wide array of candidates for particle dark matter was suggested, including neutralinos and sterile neutrinos [37,38]. However, numerous experiments have failed to find evidence for dark matter particles, and it was suggested that gravity theory should be modified [39]. Equation (3) enables the revisiting of the “dark matter” problem. Indeed, if dark matter is built from for particles for which $m<{\tilde{m}}_{0}≅\frac{k_{B}Tln2}{c^{2}}≅2.0\times 10^{-40}\;\mathrm{kg}$ takes place, they could not be registered due to the fact that they do not transform information to the surrounding media and experimental devices. Papers shedding light on the informational aspects of the “dark matter” problem are invited. 

### 2.5. The Landauer Principle and the Informational Content of the Universe

The computational capacity of the Universe was recently estimated and broadly discussed [40,41]. We involved Equation (2) for the estimation of the upper bound of the computational capacity of the Universe $\Delta I_{tot}$ (see also [28]):

(5) $$\Delta I_{tot}≅\frac{m_{tot}c^{2}}{k_{B}T}$$

where $m_{tot}≅1.5\times 10^{53}\;\mathrm{kg}$ is the mass of the observable Universe [42]. Substituting $m_{tot}≅1.5\times 10^{53}\;\mathrm{kg}$ and T=2.73 K we obtained: $\Delta I_{tot}≅3\times 10^{92}$ bits in the satisfactory vicinity of the estimation reported by Seth Lloyd [41], based on quite different considerations (when the gravitational degrees of freedom are taken into account, the estimation reported in [41] is much larger, i.e.,$\Delta I_{tot}≅10^{120}$ bits).

## 3. Conclusions

The physical roots, justification and precise meaning of the Landauer principle [1,2,3] remain obscure and were exposed to turbulent discussion recently [5,6,7,8,9,10,11,12,13,14]. We demonstrated that the Landauer principle supplies the estimation of the minimal mass of the particle allowing the recording/erasure of information within the surrounding medium at temperature *T*. Particles lighter than ${\tilde{m}}_{0}=\frac{k_{B}Tln2}{c^{2}}≅2.0\times 10^{-40}\;\mathrm{kg}$ are suggested to be incapable of transforming the information to the surrounding bodies and are well expected to be undetectable (assuming T=2.73 K, which is the temperature of the cosmic microwave background). This is true in the case when the violations of the Landauer principle are neglected [29]. All of the currently known elementary particles are heavier than ${\tilde{m}}_{0}.$ This approach is easily extended to fields. Perhaps the Landauer principle helps to explain the problem of undetectable dark matter, if we assume that dark matter is built from particles with $m<{\tilde{m}}_{0}≅\frac{k_{B}Tln2}{c^{2}}$. The Landauer principle enables the estimation of the total informational content of the Universe. Papers devoted to the relativistic and quantum aspects of the Landauer principle and its relation to the highly debatable relativistic transformation of temperatures are invited. Papers discussing the experimental verification and engineering implementations of the Landauer principle are encouraged. 
